# Downregulation of PPA2 expression correlates with poor prognosis of kidney renal clear cell carcinoma

**DOI:** 10.7717/peerj.12086

**Published:** 2021-09-06

**Authors:** Wenbiao Zhu, Huiming Jiang, Shoucheng Xie, Huanqin Xiao, Qinghua Liu, Nanhui Chen, Pei Wan, Shanming Lu

**Affiliations:** 1 Department of Pathology, Meizhou People’s Hospital, Meizhou, Guangdong, China; 2 Department of Urology, Meizhou People’s Hospital, Meizhou, Guangdong, China

**Keywords:** Kidney renal clear cell carcinoma, PPA2, Prognosis, Biomarker

## Abstract

**Background:**

Kidney renal clear cell carcinoma (KIRC) is the most common subtype of kidney cancer. Inorganic pyrophosphatase (PPA2) is an enzyme that catalyzes the hydrolysis of pyrophosphate to inorganic phosphate; few studies have reported its significance in cancers. Therefore, we aimed to explore the prognostic value of PPA2 in KIRC.

**Methods:**

PPA2 expression was detected via immunohistochemistry in a tissue chip containing specimens from 150 patients with KIRC. We evaluated the correlation between PPA2 expression, clinicopathological characteristics, and survival. Data from online databases and another cohort (paraffin-embedded specimens from 10 patients with KIRC) were used for external validation.

**Results:**

PPA2 expression was significantly lower in KIRC tissues than in normal renal tissues (*p* < 0.0001). Low expression of PPA2 was significantly associated with a high histologic grade and poor prognosis. The differential expression of PPA2 was validated at the gene and protein levels. Multivariate Cox regression analysis showed that PPA2 expression was an independent prognostic factor in patients with KIRC. Gene set enrichment analysis suggested that decreased expression of PPA2 might be related to the epithelial-mesenchymal transition in KIRC.

**Conclusions:**

Our study demonstrated that PPA2 is an important energy metabolism-associated biomarker correlated with a favorable prognosis in KIRC.

## Introduction

Kidney renal clear cell carcinoma (KIRC) is the most common subtype of kidney cancer, accounting for approximately 70%–75% of cases (Petejova & Martinek, 2016). Although the treatment of KIRC has advanced considerably in recent years, it remains a major challenge worldwide. Localized KIRC progresses to distant metastasis in more than 50% of KIRC patients, even after surgery, with a five-year survival rate of less than 12% after metastasis (Padala et al., 2020). The TNM stage is the most important prognostic factor for determining the outcome of patients with KIRC, but its predictive ability is unsatisfactory because the survival outcome can differ among patients with cancers of the same TNM stage (Martinez-Salamanca et al., 2011). Therefore, identifying effective prognostic biomarkers and therapeutic targets to improve clinical outcomes remains a hot topic in KIRC research.

Recent studies have shown that post-translational modification of molecules plays a vital role in oncogenesis and progression. Therefore, many protein enzymes, including methylases, demethylases, phosphatases, ubiquitin ligases, and deubiquitinases, have attracted increasing attention (Poondla et al., 2019; Rowbotham et al., 2018; Ruvolo, 2019; Senft, Qi & Ronai, 2018; Zeng et al., 2020). Inorganic pyrophosphatases are enzymes that catalyze the hydrolysis of inorganic pyrophosphate into two phosphate molecules. Humans possess two types of inorganic pyrophosphatases (PPA): PPA1 and PPA2. PPA1 is located in the cytoplasm and plays an important role in carcinogenesis. Wang et al. (2017) reported that PPA1 regulates the malignant potential of the tumor and the clinical outcome of patients with colon adenocarcinoma through the c-Jun N-terminal kinase (JNK) pathways. Another study showed that PPA1 expression correlated with poor survival of patients with epithelial ovarian cancer (Li et al., 2017). PPA2, encoded by *PPA2,* is located in the mitochondria, which also hydrolyzes inorganic pyrophosphate (Kennedy et al., 2016). This activity is essential for the correct regulation of mitochondrial membrane potential and mitochondrial organization and function (Guimier et al., 2016).  Mutations in *PPA2* can lead to sudden unexpected death (Phoon et al., 2020). One study showed that PPA2 expression was increased in patients with lymph node metastatic prostate cancer, suggesting that it might be a useful diagnostic marker (Pang et al., 2010). However, whether PPA2 has a similar prognostic value to PPA1 in cancer remains unknown. Therefore, we conducted this study to fill this research gap by exploring the role of PPA2 in KIRC.

## Materials & Methods

### Tissue specimens

A KIRC tissue microarray (HKidE180Su02) comprising 150 KIRC tissues and 30 normal renal specimens was purchased from Shanghai Outdo Biotech Co., Ltd (http://www.superchip.com.cn/), which was used to measure the protein expression level of PPA2 *via* immunohistochemistry (IHC). Another cohort of 10 paraffin-embedded KIRC tissues and paired normal renal tissues from patients at Meizhou People’s Hospital was used for external validation. Ethical approval was granted by the Medical Ethics Committee of Meizhou People’s Hospital (2020-CY-06) and written informed consent was obtained from all patients.

### Immunohistochemical staining and scoring

All specimens were confirmed by two independent pathologists after hematoxylin and eosin staining. The samples were first deparaffinized with dimethylbenzene and rehydrated in an ethanol-water gradient. Antigen retrieval was performed by microwave. The samples were then treated with 3% hydrogen peroxide to inhibit the activity of endogenous peroxidases, and subsequently blocked with 5% bovine serum albumin for 1 h. The samples were incubated with anti-PPA2 primary antibody (diluted 1:200, ab180859, Abcam) overnight at 4 °C. Finally, the samples were stained with 3,3′-diaminobenzidine solution for visualization after incubation with horseradish peroxidase-conjugated secondary antibody (diluted 1:10000, ab205718, Abcam) (Sun et al., 2017).

The level of PPA2 expression was evaluated based on the degree of staining and the percentage of positively stained cells. The staining degree was scored as 0 (no staining), 1 (light yellow), 2 (dark yellow), or 3 (brown). The percentage of positively stained cells was scored as 0 (<1%), 1 (1%–25%), 2 (26%–50%), 3 (51%–75%), and 4 (76%–100%) (Niu et al., 2018). We multiplied the two above scores to obtain a final IHC score ranging from 0 to 12.

### Construction and evaluation of a PPA2-based predictive nomogram

We used the identified independent prognostic factors to construct a predictive nomogram. The concordance index (C-index) and calibration plots were used to evaluate the constructive nomogram, as previously reported (Liu et al., 2019).

### Gene Set Enrichment Analysis (GSEA)

GSEA was conducted to explore the potential molecular mechanisms linking PPA2 expression to KIRC prognosis (Subramanian et al., 2005). We first divided the patients into two groups according to the median PPA2 expression level. We then identified the differentially expressed genes between the low and high expression groups and performed GSEA. The gene set permutations were conducted 1000 times for each analysis. A false discovery rate of < 0.25 and *P* value < 0.05 were considered to indicate statistical significance (Liu et al., 2020).

### Calculation of stromal and immune scores

We calculated the stromal scores and immune scores by applying the “Estimation of STromal and Immune cells in MAlignant Tumors using Expression data (ESTIMATE)” algorithm (https://bioinformatics.mdanderson.org/estimate/) to infer the fraction of stromal and immune cells and predict tumor purity for each KIRC sample in The Cancer Genome Atlas (TCGA) (Yoshihara et al., 2013). Then we downloaded the gene expression profile of TCGA-KIRC dataset from UCSC Xena platform (https://xena.ucsc.edu/) (Goldman et al., 2020). We matched the samples’ gene expression data and ESTIMATE algorithm results. Next, we divided the KIRC samples into two groups according to the median value of stromal score, immune score, and ESTIMATE score, respectively. Finally, we explored the association between PPA2 expression and algorithm results.

### Statistical analysis

All data processing and statistical analyses were performed using R software V3.6.1 (R Core Team, 2019) and SPSS V25 (SPSS Inc.) (Chan, 2018; Liang, Fu & Wang, 2019). Differential expression analysis was performed by the “limma” R package and the survival analysis was performed using the “survival” and “survminer” R packages (Ritchie et al., 2015; Wang et al., 2020). Student’s *t*-test or analysis of variance was used to evaluate the differences in PPA2 expression among the different subgroups. The paired *t*-test was used to explore the difference in PPA2 expression between the ten paraffin-embedded KIRC tissues and paired normal renal tissues. The chi-square test or Fisher’s exact test was used to investigate the relationship between PPA2 protein expression and the clinicopathological features of KIRC. The Kaplan–Meier method and log-rank test were used for the survival analyses. The Cox regression model was used for univariate and multivariate survival analyses. For all analyses, a *P*-value < 0.05 was regarded as statistically significant.

## Results

### Clinicopathological characteristics

A total of 150 KIRC patients whose specimens were present in the tissue microarray were enrolled in this retrospective study. All patients underwent nephrectomy or partial nephrectomy. The median age was 57 years (range, 24–83 years). Sixty-three patients (42%) were aged above 60 years. Specimens from 103 patients (68.7%) were histologically graded as relatively well-differentiated (G1/2), whereas those from the remaining 47 patients (31.3%) were relatively poorly differentiated (G3/4). Most of the patients (138 cases, 92%) had localized tumors of American Joint Committee on Cancer (AJCC) stage I/II, whereas the other 12 patients (8%) had AJCC stage III/IV tumors. The detailed clinicopathological information of the enrolled patients is summarized in Table 1.

### PPA2 expression profiles and their relationship with KIRC clinicopathological parameters

**Figure 1:**
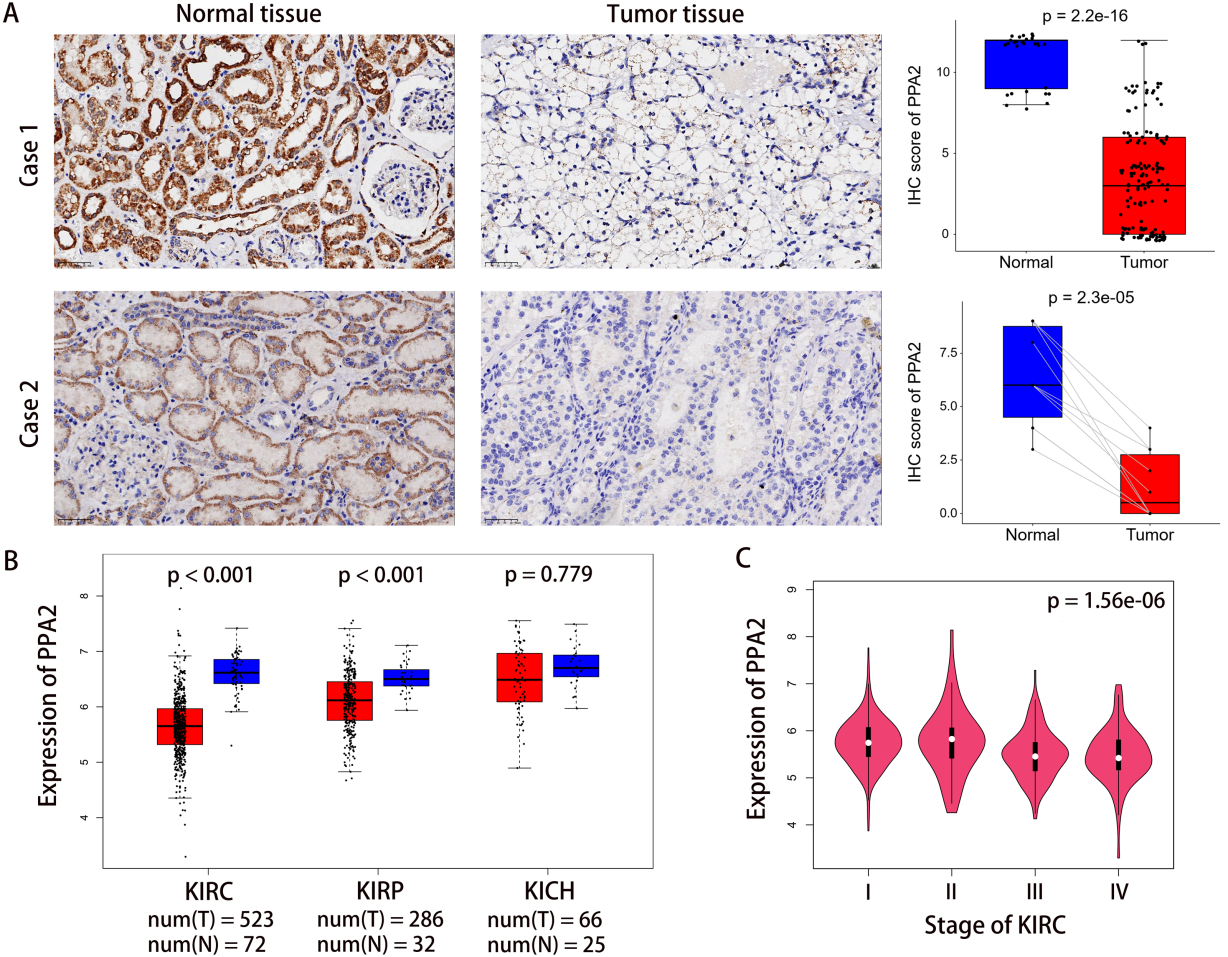
PPA2 expression is significantly lower in KIRC tissues than in normal tissues. (A) Representative images of immunohistochemically-stained tumor tissues and normal tissues. Case 1 was derived from a tissue microarray which contained 150 tumor tissues and 30 normal tissues, whereas Case 2 represents another cohort from Meizhou People’s Hospital which contained 10 tumor tissues and paired normal tissues. (B) Differential PPA2 expression in TCGA KIRC cohort. (C) PPA2 expression was significantly different among patients with different cancer stage. B and C represent the results of analyses carried out on the GEPIA2 platform. KIRC, kidney renal clear cell carcinoma; KIRP, Kidney renal papillary cell carcinoma; KICH, Kidney chromophobe; IHC, immunohistochemistry.

Immunohistochemical staining on the tissue microarray showed that PPA2 was primarily expressed in the cytoplasm of KIRC cells, and that the protein expression level of PPA2 in tumor tissues was significantly lower than that in normal renal tissues (Fig. 1A). To corroborate our results, we first validated the differences in *PPA2* expression at the gene level using data from TCGA on the Gene Expression Profiling Interactive Analysis (GEPIA2) platform (Tang et al., 2019) (http://gepia2.cancer-pku.cn). This analysis showed that *PPA2* was expressed at lower levels not only in KIRC, but also in two other common kidney cancer types (Fig. 1B). We then used another cohort containing ten paraffin-embedded KIRC tissues and paired normal renal tissues to validate these results at the protein level. This analysis confirmed that PPA2 expression was lower in the KIRC tissues (Fig. 1A).

**Table 1:** Association between PPA2 expression and clinicopathologic characteristics in KIRC.

**Characteristic**	***n* (%)**	**PPA2 expression (%)**		*p*-value
		**High**	**Low**	
Total	150 (100)	70 (46.7)	80 (53.3)	
Age				0.411
<60 years	87 (58.0)	38 (54.3)	49 (61.3)	
≥ 60 years	63 (42.0)	32 (45.7)	31 (38.8)	
Gender				0.475
Male	107 (71.3)	52 (74.3)	55 (68.8)	
Female	43 (28.7)	18 (25.7)	25 (31.2)	
Grade				0.014
G1/2	103 (68.7)	41 (58.6)	62 (77.5)	
G3/4	47 (31.3)	29 (41.4)	18 (22.5)	
N stage				0.248
N0	147 (98.0)	70 (100)	77 (96.2)	
N1	3 (2.0)	0 (0)	3 ( 3.8)	
AJCC stage				0.141
I/II	138 (92.0)	67 (95.7)	71 (88.8)	
III/IV	12 (8.0)	3 ( 4.3)	9 (11.2)	

**Notes.**

Abbreviations KIRC kidney renal clear cell carcinoma AJCC American Joint Committee on Cancer

The 150 patients enrolled in our study were divided into high and low PPA2 expression groups based on their median expression values. Details of the association between PPA2 expression levels and clinicopathological features of KIRC are shown in Table 1. PPA2 expression was lower in tumors with histologic grade G3/4 than in those with grade G1/2 (*p* < 0.014). However, there was no significant correlation between PPA2 expression and patient AJCC stage, possibly due to the small sample size of patients with stage III/IV KIRC. Considering the importance of the AJCC stage for outcome prediction, we further explored the association between PPA2 expression and AJCC stage using data from the TCGA KIRC cohort (including 523 patients with KIRC) on the GEPIA2 platform. We found that PPA2 expression was also significantly different among patients with different AJCC stages (*p* < 0.001, Fig. 1C).

### Prognostic potential of PPA2 in KIRC

**Figure 2:**
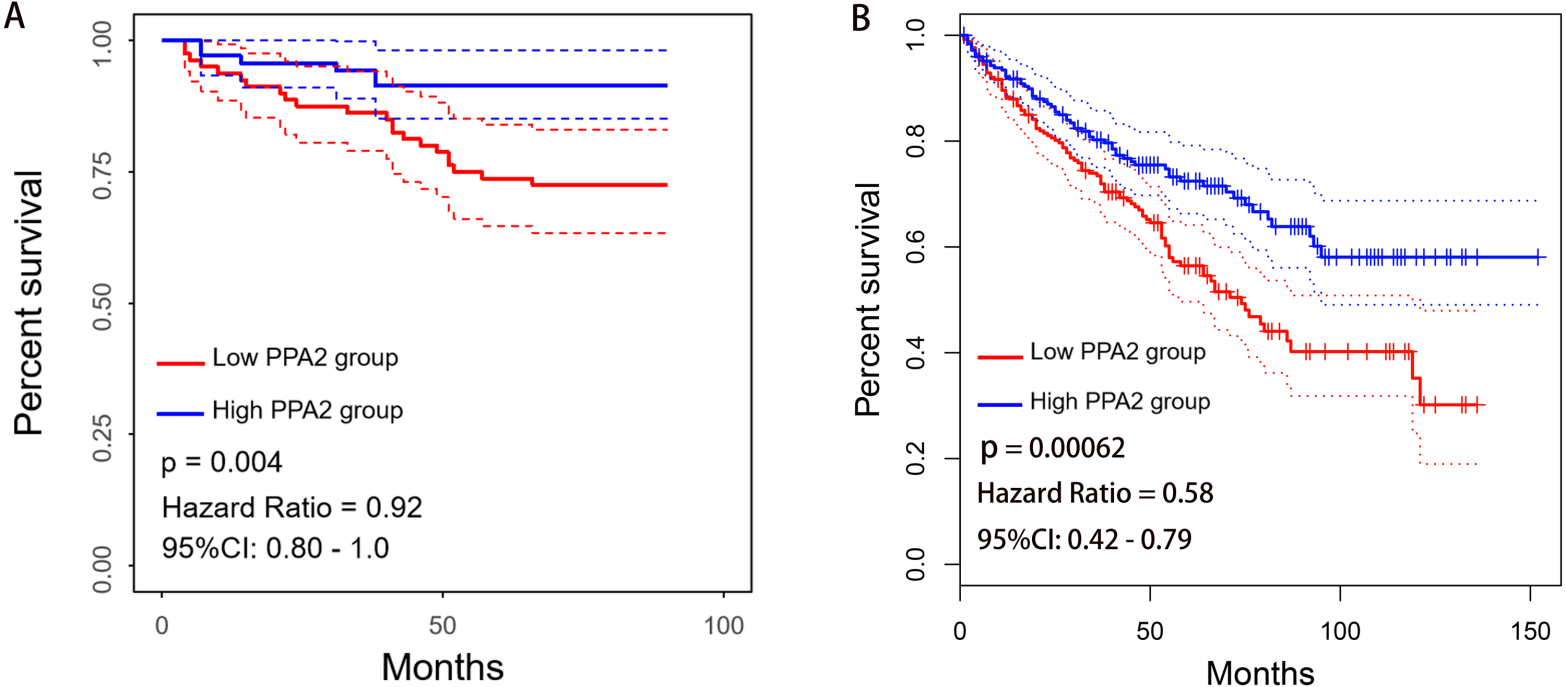
Association between PPA2 expression and the OS of KIRC patients. (A) Low PPA2 expression at the protein level is significantly correlated with poor OS. (B) Validation of the correlation between PPA2 expression and OS on the GEPIA2 platform. KIRC, kidney renal clear cell carcinoma; OS, overall survival.

Kaplan–Meier survival analysis revealed that patients with low PPA2 expression exhibited worse overall survival (OS) than those with high PPA2 expression (*p* = 0.004, Fig. 2A). To confirm these results, we further explored the prognostic potential of PPA2 in a pan-cancer dataset (including 33 cancer types and 9,486 patients in total) from TCGA using the GEPIA2 platform. Interestingly, PPA2 also displayed prognostic value in several other cancers, including breast invasive carcinoma, colon adenocarcinoma, kidney renal papillary cell carcinoma, brain lower grade glioma, and uveal melanoma (Fig. 2B, Table 2).

**Table 2:** Kaplan-Meier survival analysis for PPA2 in pan-cancer (33 kinds of cancer from TCGA).

Abbreviation	Detail	Number of patients	HR	Log-rank test *p*-value
ACC	Adrenocortical carcinoma	76	0.82	0.62
BLCA	Bladder Urothelial Carcinoma	402	0.96	0.8
BRCA	Breast invasive carcinoma	1070	1.6	0.0058
CESC	Cervical squamous cell carcinoma and endocervical adenocarcinoma	292	1	0.88
CHOL	Cholangio carcinoma	36	1.4	0.48
COAD	Colon adenocarcinoma	270	0.49	0.0042
DLBC	Lymphoid Neoplasm Diffuse Large B-cell Lymphoma	46	1.6	0.49
ESCA	Esophageal carcinoma	182	1.1	0.82
GBM	Glioblastoma multiforme	162	0.71	0.056
HNSC	Head and Neck squamous cell carcinoma	518	1	0.73
KICH	Kidney Chromophobe	64	3.1	0.15
KIRC	Kidney renal clear cell carcinoma	516	0.58	0.00062
KIRP	Kidney renal papillary cell carcinoma	282	0.44	0.012
LAML	Acute Myeloid Leukemia	106	1.2	0.6
LGG	Brain Lower Grade Glioma	514	1.5	0.021
LIHC	Liver hepatocellular carcinoma	364	0.95	0.77
LUAD	Lung adenocarcinoma	478	1.2	0.14
LUSC	Lung squamous cell carcinoma	482	1	0.89
MESO	Mesothelioma	82	0.85	0.5
OV	Ovarian serous cystadenocarcinoma	424	0.93	0.53
PAAD	Pancreatic adenocarcinoma	178	1.2	0.34
PCPG	Pheochromocytoma and Paraganglioma	182	1.7	0.55
PRAD	Prostate adenocarcinoma	492	1.7	0.41
READ	Rectum adenocarcinoma	92	0.41	0.085
SARC	Sarcoma	262	1	0.91
SKCM	Skin Cutaneous Melanoma	458	0.91	0.5
STAD	Stomach adenocarcinoma	384	1.1	0.46
TGCT	Testicular Germ Cell Tumors	138	0.34	0.33
THCA	Thyroid carcinoma	510	1.4	0.48
THYM	Thymoma	118	5.5	0.074
UCEC	Uterine Corpus Endometrial Carcinoma	172	0.68	0.29
UCS	Uterine Carcinosarcoma	56	1.1	0.83
UVM	Uveal Melanoma	78	4.4	0.0038

### Independent prognostic value of PPA2 in KIRC

**Figure 3:**
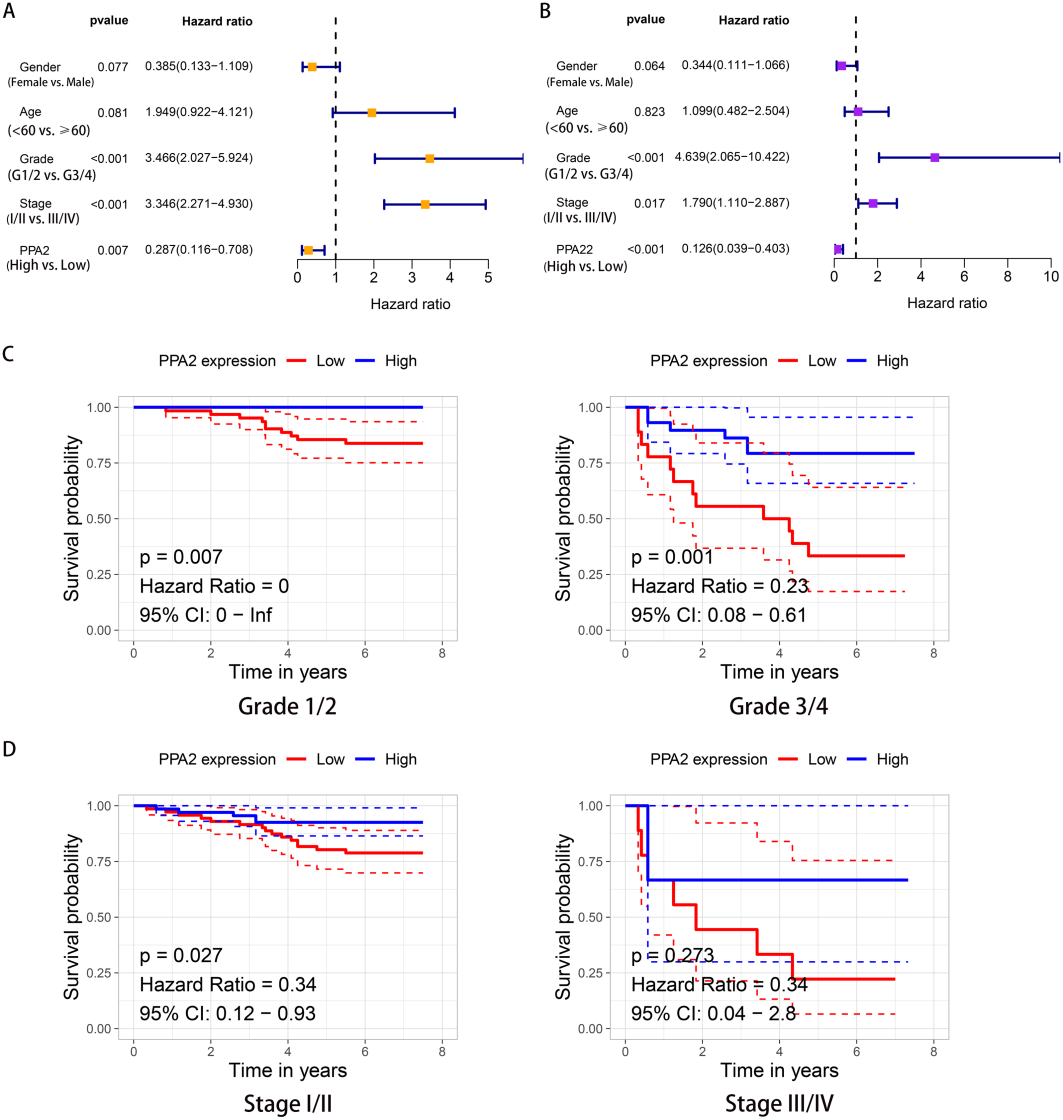
Independent prognostic value of PPA2 in KIRC. (A) Forest plot showing the results of univariate Cox regression analysis. (B) Forest plot showing the results of multivariate Cox regression analysis. (C) Kaplan–Meier survival analysis for PPA2 expression stratified by histologic grade. (D) Kaplan–Meier survival analysis for PPA2 expression stratified by stage. KIRC, kidney renal clear cell carcinoma.

Cox survival analysis revealed a significant survival difference between the high and low PPA2 expression groups in both univariate (hazard ratio [HR], 0.287; 95% confidence interval CI [0.116–0.708]; *p* = 0.007) and multivariate (HR, 0.126; 95% CI [0.039–0.403]; *p* < 0.001) analyses (Figs. 3A, 3B). PPA2 expression, histologic grade, and AJCC stage showed independent prognostic values in KIRC. Moreover, we performed further stratified analysis based on independent prognostic factors, histologic grade and AJCC stage. This analysis showed that patients with low PPA2 protein expression had poorer OS than those with high PPA2 protein expression when bearing tumors of histologic grade 1/2 (*p* = 0.007), grade 3/4 (*p* = 0.001), or AJCC stage I/II (*p* = 0.027), but not stage III/IV (*p* = 0.273) (Figs. 3C, 3D).

### Construction and evaluation of a predictive nomogram

**Figure 4:**
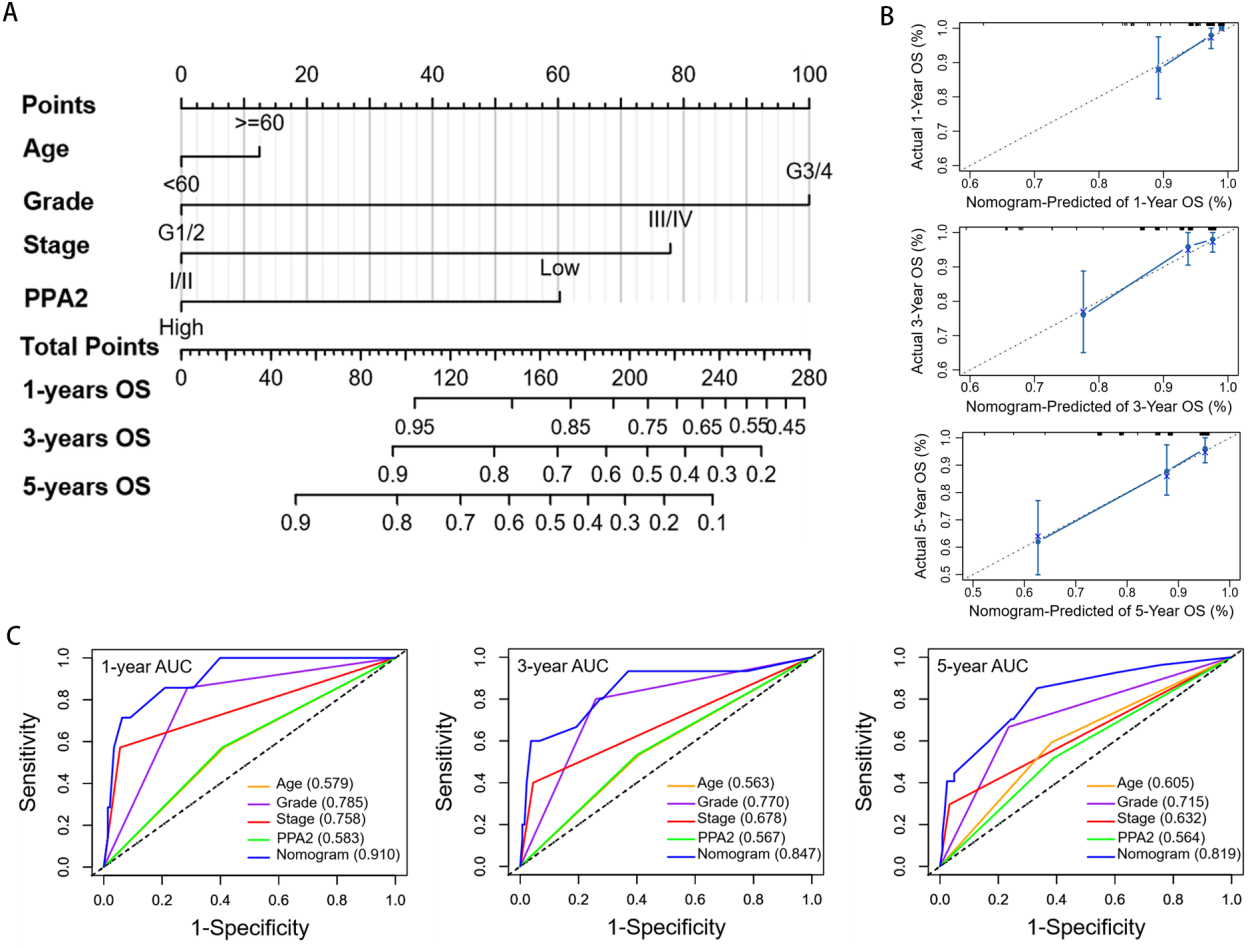
Construction andevaluation of a nomogram predicting the overall survival of KIRC patients. (A) Nomogram for predicting overall survival. (B) Calibration plots denoting the agreement between the nomogram-predicted survival rate and the actual survival rate. Actual survival is shown on the Y axis, and nomogram-predicted survival is shown on the X axis. The solid line is very close to the 45-degree dotted line, denoting the good predictive ability of the nomogram. (C) Comparison of the time-dependent receiver operating characteristic curves of the nomogram with those relative to other independent prognostic factors of overall survival of KIRC patients. AUC, area under the curve; KIRC, kidney renal clear cell carcinoma.

Nomograms are widely used to predict the outcomes of cancer patients (Iasonos et al., 2008). The C-index and calibration plots are often used to investigate the discrimination ability and calibration of nomograms (Fenlon et al., 2018; Longato, Vettoretti & Di Camillo, 2020). In this study, patient age, histologic grade, AJCC stage, and PPA2 expression were used to construct a nomogram to predict the 1-year, 3-year and 5-year OS probability of KIRC patients, which could provide helpful information for individualized clinical evaluation and therapeutic strategies (Fig. 4A). The C-index of our nomogram was 0.795, which was significantly higher than that of any of the other independent prognostic factors (age, 0.582; histologic grade, 0.695; AJCC stage, 0.623; and PPA2 expression, 0.569). The calibration plot indicated that the 1-year, 3-year, and 5-year OS probabilities predicted by our nomogram were consistent with the actual outcomes (Fig. 4B). Furthermore, the area under the curve of our nomogram for the 1-year, 3-year, and 5-year OS of KIRC patients was 0.910, 0.847, and 0.819, respectively, displaying greater values than those found for any prognostic factor, including age, histologic grade, AJCC stage, and PPA2 expression (Fig. 4C). Taken together, these results suggest that our nomogram could accurately predict the OS of KIRC patients with KIRC.

### GSEA

**Figure 5:**
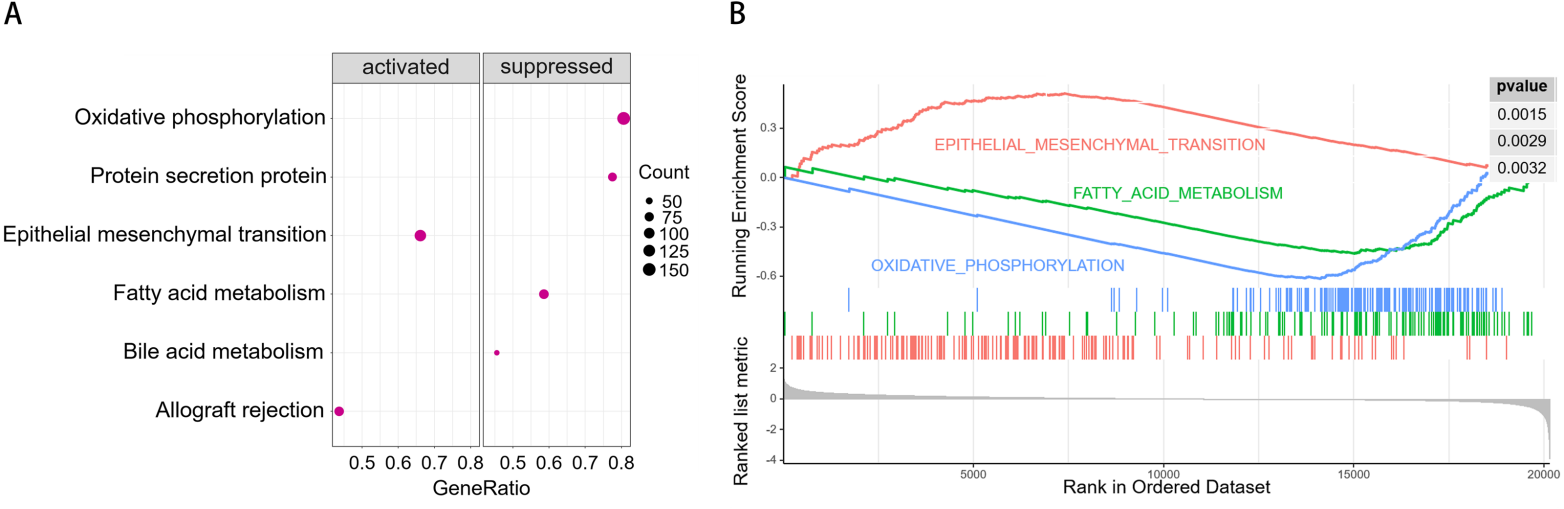
Results of gene set enrichment analysis (GSEA). (A) Bubble chart showing the top activated and suppressed signaling pathways in KIRC tissues with decreased PPA2 expression. (B) Enrichment plot showing three selected representative pathways. KIRC, kidney renal clear cell carcinoma.

To understand the potential molecular mechanism underlying the prognostic value of PPA2 in KIRC, we conducted GSEA using transcriptome data from the TCGA KIRC cohort, downloaded from the Xena platform of the University of California, Santa Cruz (Goldman et al., 2020) (https://xena.ucsc.edu/). The results of GSEA suggested that the epithelial-mesenchymal transition (EMT) and allograft rejection pathways were possibly activated in patients with low PPA2 expression, whereas the pathways of oxidative phosphorylation, protein secretion, bile acid metabolism and fatty acid metabolism were possibly suppressed (Fig. 5).

### Differential PPA2 expression between different stromal/immune score groups

**Figure 6:**
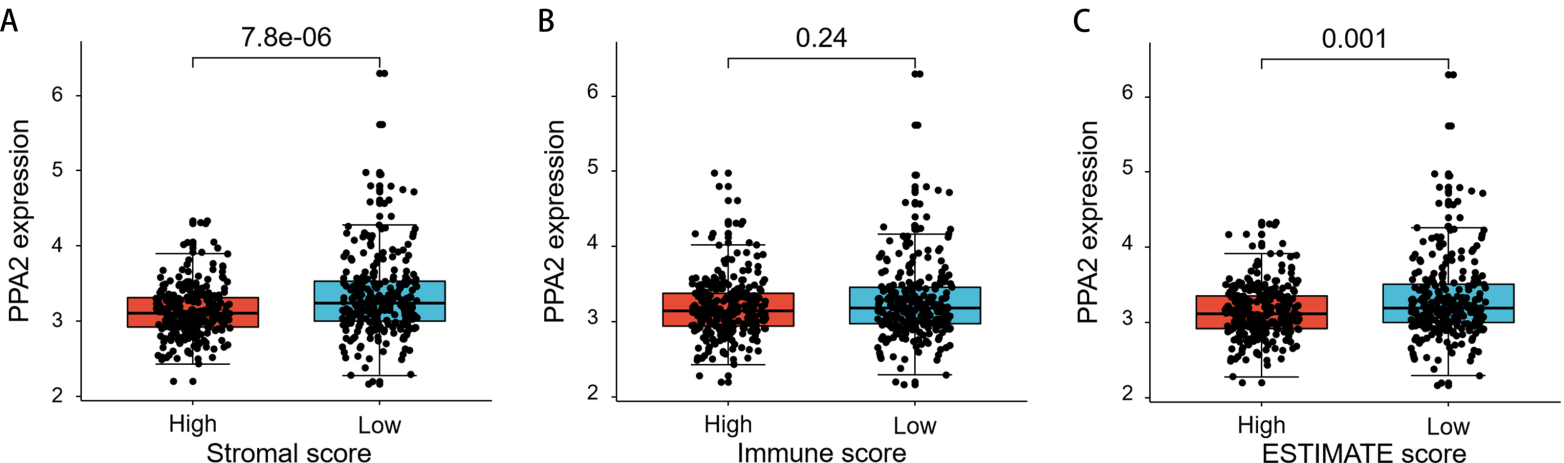
The differential PPA2 expression between different stromal/immune score groups. (A) PPA2 was significantly upregulated in samples with low stromal scores than that in samples with high stromal scores. (B) There is not significant difference of PPA2 expression between different immune score groups. (C) PPA2 was significantly upregulated in samples with low ESTIMATE scores than that in samples with high ESTIMATE scores.

The ESTIMATE algorithm is helpful for us to understand the landscape of stromal and immune cells in the tumor microenvironment. The higher stromal/immune scores mean more stromal/immune cells in tumor microenvironment, whereas higher ESTIMATE scores mean lower tumor purity (less tumor cells). Our study showed that PPA2 was significantly higher expressed in samples with lower stromal scores or ESTIMATE scores (Fig. 6). It suggested that PPA2 might be expressed highest in tumor cells.

## Discussion

KIRC treatment remains a major challenge for urologists because of its high mortality rate. One key reason for this poor outcome is the lack of effective prognostic and therapeutic biomarkers for this disease (Hsieh et al., 2017; Williamson, Taneja & Cheng, 2019). Thus, it is necessary to identify effective biomarkers to distinguish patients at risk of poor survival. Considering that energy metabolism reprogramming is a hallmark of cancer (Hanahan & Weinberg, 2011) and that phosphorylation/dephosphorylation is critical in biological processes, we explored the clinical significance and possible role of PPA2, an inorganic pyrophosphatase, in KIRC.

PPA2 is highly similar to other members of the inorganic pyrophosphatase family, catalyzing the hydrolysis of pyrophosphate to inorganic phosphate, which is essential for cellular phosphate metabolism and energy metabolism, and is also required in many biological processes including the synthesis of RNA, DNA, proteins, polysaccharides, and lipids (Guimier et al., 2016). Pang et al. (2010) used differential in-gel electrophoresis-based proteomics analysis and found that PPA2 was overexpressed in patients with lymph node metastatic prostate cancer, suggesting the potential diagnostic value of PPA2. Uzozie et al. (2017) also found that PPA2 expression was upregulated in colorectal adenomas using a targeted proteomics approach. However, the prognostic value of PPA2 in cancer has rarely been reported. In this study, by performing IHC in samples from 150 KIRC patients, we found that PPA2 expression was significantly lower in tumor tissues compared than in normal renal tissues, and that lower PPA2 expression was significantly correlated with patient OS. We also validated these findings in another independent cohort consisting of 10 KIRC patients, as well as using data from the TCGA KIRC cohort. Furthermore, this study showed that lower PPA2 expression was related to an unfavorable higher histologic grade and advanced stage of KIRC. Multivariate Cox regression analysis showed that PPA2, higher histologic grade, and advanced stage played independent prognostic roles in KIRC. In addition, we performed stratified analysis according to histologic grade and stage, and confirmed that the high PPA2 expression group had a significantly better OS. However, we also noted no survival differences among KIRC patients with stage 3/4 cancer. This insignificant correlation might be due to the small sample size of this group of patients (only 12 cases in total). In addition, we established a nomogram based on PPA2 expression, which could accurately predict the OS of patients with KIRC. These results suggest that PPA2 is a novel potential prognostic biomarker for KIRC.

The close association between PPA2 expression and the clinical characteristics of KIRC patients prompted us to further explore the possible molecular mechanism of PPA2 activity in KIRC. Interestingly, our GSEA results revealed that decreased PPA2 expression was possibly associated with activated EMT and suppressed oxidative phosphorylation and fatty acid metabolism. The suppression of oxidative phosphorylation is conducive to glycolysis, which is the most important mechanism of energy production in cancer. The suppression of fatty acid metabolism might cause the accumulation of fatty acids and result in the reprogramming of fatty acid metabolism (Wettersten et al., 2017). EMT is an important characteristic of tumor metastasis (Brabletz et al., 2018). Notably, many studies have confirmed a close association between EMT and fatty acid metabolism (Giudetti et al., 2019; Jiang et al., 2020; Li et al., 2019). However, it is still unclear whether PPA2 could affect the fatty acid metabolism and EMT or not. On the other hand, accumulating evidence has demonstrated that tumor microenvironment plays a crucial role in cancer progression, survival, and other biological characteristics, including reprogramming of fatty acid metabolism and EMT (Aggarwal et al., 2021; Drak Alsibai & Meseure, 2018; Lyssiotis & Kimmelman, 2017). Our study also showed that PPA2 expression was significantly differential between groups with different stromal scores or ESTIMATE scores, suggesting that the tumor microenvironment might affect PPA2 expression.

Although our study provides the first evidence for the potential value of PPA2 in KIRC prognosis, several limitations are worth discussing. First, our study was a retrospective analysis, for which selection bias is inevitable. Second, information on some important pathological features such as lymphovascular invasion, sarcomatoid change, and tumor necrosis, which might have prognostic value, was unavailable. Finally, the molecular mechanism underlying PPA2 activity in KIRC was explored mainly based on the data of TCGA-KIRC cohort, more KIRC samples from real world and rigorous wet lab experiments were needed to investigate the potential mechanism.

## Conclusions

The present study revealed that PPA2 is an important energy metabolism-associated biomarker that has a favorable impact on prognosis in KIRC.

## Supplemental Information

10.7717/peerj.12086/supp-1 Supplemental Information 1 Raw data of PPA2 expression of specimens in chip

10.7717/peerj.12086/supp-2 Supplemental Information 2 Raw data of PPA2 expression of specimens from Meizhou People’s Hospital

10.7717/peerj.12086/supp-3 Supplemental Information 3 Clinicopathological characteristics of KIRC patients

10.7717/peerj.12086/supp-4 Supplemental Information 4 R code

10.7717/peerj.12086/supp-5 Supplemental Information 5 Factor translation Codebook to convert numbers to their respective factors.

## Abbreviations

KIRC: kidney renal clear cell carcinoma
C-index: concordance index
PPA2: inorganic pyrophosphatase
IHC: immunohistochemistry
GSEA: gene set enrichment analysis
ESTIMATE: Estimation of STromal and Immune cells in MAlignant Tumors using Expression data
OS: overall survival
TCGA: The Cancer Genome Atlas
AJCC: American Joint Committee on Cancer
EMT: epithelial-mesenchymal transition

## Additional Information and Declarations

### Microarray Data Deposition

The following information was supplied regarding the deposition of microarray data: The tissue microarray (HKidE180Su02) was purchased from Shanghai Outdo Biotech Co., Ltd (http://www.superchip.com.cn/).

### Data Availability

The following information was supplied regarding data availability: The raw data are available in the Supplemental Files. The TCGA-KIRC dataset is available at Xena under “GDC TCGA Kidney Clear Cell Carcinoma (KIRC)”. https://xena.ucsc.edu/.
